# Bacterial ι-carbonic anhydrase: a new active class of carbonic anhydrase identified in the genome of the Gram-negative bacterium *Burkholderia territorii*

**DOI:** 10.1080/14756366.2020.1755852

**Published:** 2020-04-21

**Authors:** Sonia Del Prete, Alessio Nocentini, Claudiu T. Supuran, Clemente Capasso

**Affiliations:** a Department of Biology, Agriculture and Food Sciences, Institute of Biosciences and Bioresources, CNR, Napoli, Italy; b Department of NEUROFARBA, University of Florence, Section of Pharmaceutical and Nutraceutical Sciences, Firenze, Italy

**Keywords:** Carbonic anhydrase, bacterial CA, *Burkholderia territorii*, iota-CA, hydratase activity, catalytic ion cofactor

## Abstract

The carbonic anhydrases (CAs, EC 4.2.1.1) catalyse a simple but physiologically crucial reversible reaction, the carbon dioxide hydration with the production of bicarbonate and protons. In the last years, and especially, to the rapid emergence of the bacterial antibiotic resistance that is occurring worldwide, the understanding of the function of bacterial CAs has increased significantly. Recently, a new CA-class (ι-CA) was discovered in the marine diatom *T. pseudonana*. It has been reported that bacterial genomes may contain genes with relevant homology to the diatom ι-class CA. Still, the catalytic activity of the enzyme encoded by the gene was not investigated. Thus, herein, for the first time, we cloned, expressed, and purified the recombinant bacterial ι-CA (acronym BteCAι) identified in the genome of *Burkholderia territorii*. The recombinant BteCAι resulted in a good catalyst for the hydration of CO_2_ to bicarbonate and protons, with a k_cat_ of 3.0 × 10^5^ s ^−1^ and k_cat_/K_M_ of 3.9 × 10^7^ M ^−1^ s ^−1^, and is also sensitive to inhibition by the sulphonamide acetazolamide. Furthermore, with the aid of the protonography, it has been demonstrated that BteCAι can be present as a dimer. This result is corroborated by the construction of a molecular model of BteCAι, which showed that the enzyme is formed by two equivalent monomers having a structure similar to a butterfly.

## Introduction

The metalloenzyme carbonic anhydrases (CAs, EC 4.2.1.1) catalyse a simple but physiologically crucial reversible reaction, the carbon dioxide hydration with the production of bicarbonate and protons (CO_2_ + H_2_O ⇄ HCO_3_^–^ + H^+^)^1^. The CO_2_ hydration reaction is catalysed at very high rates, with a pseudo-first-order kinetic constant (k_cat_) ranging from 10^4^ to 10^6^ s^−1^ ^2^^,^^3^. In Eukaryotes, CAs are involved in the transport and supply of CO_2_ or HCO_3_^–^, pH homeostasis, secretion of electrolytes, biosynthetic processes, and photosynthesis^4^^,^^5^. Since the CA hydratase/dehydratase activity contributes to numerous physiological functions involving dissolved inorganic carbon, CAs are crucial biomolecules in many physiological and pathological conditions in all types of organisms^6^. In the last years, and especially, to the rapid emergence of the bacterial antibiotic resistance that is occurring worldwide, the understanding of the function of bacterial CAs has increased significantly^7^^,^^8^. CAs are essential for the survival of the microbes and their pathogenicity and virulence^9^^,^^10^. It has been demonstrated *in vivo* that the bacterial growth at ambient CO_2_ concentration is dependent on CA activity for several species, such as in the organisms discussed below^11^^,^^12^. Thus, CAs encoded in the genome of *Helicobacter pylori,* are essential for the acid acclimatisation of the pathogen within the stomach^13–15^; *Vibrio cholerae* uses CAs as a system to colonise the host since CAs are involved in the production of sodium bicarbonate, which induces cholera toxin expression^16^; *Brucella suis* needed functional CAs for growing^17–20^; *β*-CA from *Salmonella enterica* is highly expressed during the bacterial infection^21^. Finally, the deletion of the gene encoding for the *β*-CA in *Pseudomonas aeruginosa* (psCA1) impaired virulence of the pathogen by reducing calcium salt depositions^22^. Starting from 1920, when was observed for the first time the CA activity in hemolyzed blood^23^, the knowledge of the enzymes responsible for that activity, i.e., the CA, has extensively been improved. Briefly, in all the living organisms, eight genetically distinct classes, named with the Greek letters, represent the CA superfamily: *α*-, *β*-, γ-, *δ*-, ζ-, *η*, *θ*, and ι^7^^,^^9^. The last three classes were only recently discovered^24^^,^^25^. All the catalytically active CAs contain, independently of the genetic groups, a metal ion cofactor, which is necessary for enzyme catalysis^7^^,^^9^^,^^26^^,^^27^. The *α*-, *β*-, *δ*-, *η*- and, perhaps *θ*-CAs use as catalytic metal the Zn^2+^ ion; γ-CAs are Fe^2+^ enzymes, but they are active with bound Zn(II) or Co^2+^ ions too^28–35^. ζ-CAs are cambialistic enzymes, which are active both with Cd^2+^ or Zn^2+^ ^2^^,^^36^^,^^37^. Unexpectedly, the last identified ι-CA, which is encoded in the genome of the marine diatom *Thalassiosira pseudonana*, prefers Mn^2+^ to Zn^2+^ as a cofactor^24^. In the CA active site, the metal is coordinated by three amino acid residues and, the fourth ligand is a water molecule/hydroxide ion acting as the nucleophile in the catalytic enzyme cycle^3^^,^^7^^,^^26^^,^^27^^,^^38^^,^^39^. The metal coordination is rather variegated among the CA-classes since in the *α*-, γ-, *δ*- and, probably, *θ*-classes the ion cofactor is coordinated by three His residues; by one His, and two Cys residues in *β*- and ζ-CAs; by two His and one Gln residues in the *η*-class^40^. In the diatom ι-CAs the putative residues able to coordinate the Mn(II) are probably two His, one Asp and one Glu, but this has not yet been clearly proved by any biophysical technique^24^. The CA classes are assembled with different folding and structures. *α*-CAs are usually active as monomers or dimers; *β*-CAs are active only as dimers, tetramers, or octamers, whereas γ-CAs must be trimers for accomplishing their physiological function^29^^,^^30^^,^^33^^,^^41^. *θ*-CAs seem to have an X-ray crystal structure very similar to the *β*-CAs^42^. The crystal structure of ζ-CA showed three slightly different active sites on the same polypeptide chain^37^. X-ray crystal structures of *δ*-, *η*-, and ι-CAs are not yet available. Intriguing, *α*-, *η*-, θ- and ι-CAs were reported to catalyse the hydrolysis of esters/thioesters, while no esterase activity was detected for the other CA families^3^^,^^24^^,^^43^. Finally, the CA-classes are differently distributed among the living organisms. For example, CAs present in mammals belong to *α*-class^44^^,^^45^, plants and algae have *α*-, *β*-, γ-, *δ*- and *θ*-classes; fungi encode for *α*- and *β*-CAs; protozoa for *α*-, *β*- and/or *η*-CAs^9^. In metazoans, the *α*-CAs are the predominant enzymes showing CO_2_ hydratase activity^46^^,^^47^. *α*-, *β*-, and γ-CAs are the typical classes present in Bacteria^7^^,^^26^^,^^27^^,^^48–51^. In 2019, Gontero et al. discovered the ι-CA class in the diatom *T. pseudonana*, as mentioned above^24^. They also reported that bacterial genomes may contain genes with relevant homology to the diatom ι-class CA, but such proteins were never characterised so far. However, most of these new bacterial sequences were annotated in the data bank as oxidoreductases. In the only work on ι-CAs published so far, the authors did not investigate if bacterial such enzymes possess a catalytic activity similar to the one reported for the diatom enzyme^24^.

Thus, herein, for the first time, we cloned, expressed, and purified the recombinant bacterial ι-CA (acronym BteCAι) identified in the genome of *Burkholderia territorii*, a Gram-negative bacterium found in soil and water, which is often resistant to common antibiotics^52^.

## Materials and methods

### Chemicals and instruments

IPTG and antibiotic were purchased from Sigma, Affinity column (His-Trap FF), molecular weight markers from GE Healthcare. All other chemicals used in this study were of reagent grade. AKTA-Prime purification system was purchased by GE Healthcare. The SX20 Stopped-Flow was obtained by the AppliedPhotophysics, while SDS–PAGE apparatus was procured by BioRAD.

### Enzyme cloning, expression and purification

The synthetic *B. territorii* gene encoding for the BteCAι was cloned into the expression vector pET100D-Topo/BteCAι and used to transform the Competent Escherichia coli BL21 (DE3) codon plus cells (Agilent) as reported by Del Prete et al.^53^. The cellular culture was induced with Isopropyl *β*-D-1-thiogalactopyranoside (IPTG) to overexpress the recombinant BteCAι. After the growth, the cells were harvested and disrupted by sonication. Cellular extract was purified using a nickel affinity column (His-Trap FF). HisTrap column (1.0 mL) was equilibrated with 20 mL equilibration buffer (50 mM Tris, 20 mM imidazole and 150 mM sodium chloride, pH 7.5) at 1 mL/min. The supernatant from the cellular lysate was loaded onto the column at 1.0 mL/min, connected with AKTA Prime. The recombinant BteCAι was eluted from the column with a flow of 0.5 mL/min and the elution buffer composed of 50 mM Tris, 500 mM imidazole and 300 mM sodium chloride, pH 7.5. The recovered BteCAι was 90% pure. The protein quantification was carried out by Bradford method (BioRAD)^54^.

### Sds-Page, protonography and Western blot

A 12% Sodium Dodecyl Sulfate-polyacrylamide gel electrophoresis (SDS-Page) prepared as described by described by Laemmli^55^ was running loading on the gel the recovered BteCAι from the affinity column. The gel was stained with Coomassie Brilliant Blue-R. To perform the protonography, wells of 12% SDS-PAGE gel were loaded with BteCAι and bCA mixed with loading buffer without 2-mercaptoethanol and without boiling the samples, in order to avoid protein denaturation. Moreover, aliquots of the purified BteCAι were mixed with a loading solution buffer (LSB) containing different concentrations of SDS, ranging from 1.0 to 0.1%. The gel was run at 150 V until the dye front ran off the gel. Following the electrophoresis, the 12% SDS-PAGE gel was subject to protonography to detect the yellows bands due to the hydratase activity on the gel as described by Capasso and co-workers^56–59^. Besides, the BteCAι subjected to a 12% (w/v) SDS-PAGE and followed by electrophoretic was transferred to a PVDF membrane with transfer buffer (25 mM Tris, 192 mM glycine, 20% methanol) using Trans-Plot SD Cell (Bio-Rad, Hercules, CA, USA). His-Tag Western blot was carried out using the Pierce Fast Western Blot Kit (Thermo Scientific,Waltham, MA, USA). Blotted membrane had been placed in the wash blot solution Fast Western 1 Wash Buffer to remove transfer buffer. Primary Antibody Working Dilution was added to the blot and incubated for 30 min at room temperature (RT) with shaking. Afterwards, the blot was removed from the primary antibody solution and incubated for 10 min with the FastWestern Optimised HRP ReagentWorking Dilution. Subsequently, the membrane was washed two times in about 20 mL of FastWestern 1 Wash Buffer. Finally, the membrane was incubated with the detection reagent working solution and incubated for 1.0 min at room temperature and then developed with X-ray film.

### Assay for carbonic anhydrase using CO_2_ as substrate

CA activity assay was performed as described by Capasso et al.^60^. Briefly, the assay was based on the monitoring of pH variation due to the catalysed conversion of CO_2_ to bicarbonate. Bromothymol blue was used as the indicator of pH variation and the assay was performed at 0 °C. The CO_2_-satured solution was used as substrate. To test the activity of carbonic anhydrase, 1.0 mL of 25 mM Tris, pH 8.3, containing bromothymol blue as a dye (to give a distinct and visible blue colour) was added to two test tubes chilled in an ice bath. An appropriate amount of the enzyme solution was added to one tube, and an equivalent amount of buffer was added to the second tube as control. One millilitre of CO_2_ solution was added, and the time required for the solution to change from blue to yellow was recorded (transition point of bromothymol blue is pH 6.0–7.6). The time required for the colour change is inversely related to the quantity of enzyme present in the sample. Wilbur-Anderson units were calculated according to the following definition: One Wilbur-Anderson unit (WAU) of activity is defined as (T_0_ − T)/T, where T_0_ (uncatalyzed reaction) and T (catalysed reaction) are recorded as the time (in seconds) required for the pH to drop from 8.3 to the transition point of the dye in a control buffer and in the presence of enzyme, respectively. The enzyme restoring activity with the metal ion was determined on samples aliquots kept overnight in a solution containing 150 mM NaCl and 20 mM Tris-HCl, pH 8.0, with or without 5 mM EDTA. EDTA-treated samples were mixed with 10 mM of CaCl_2_, MnCl_2_, and ZnCl_2_ and incubated at room temperature for 45 min before to performing the CO_2_ hydratase assay.

### Determination of the kinetic parameters and inhibition constants

The CO_2_ hydration activity performed by the BteCAι was monitored using an Applied Photophysics stopped-flow instrument^61^. Phenol red (at a concentration of 0.2 mM) was used as indicator, working at the absorbance maximum of 557 nm, with 20 mM TRIS (pH 7.5) as buffer, and 20 mM NaClO_4_ (for maintaining constant the ionic strength), following the initial rates of the CA-catalysed CO_2_ hydration reaction for a period of 10–100 s. To determine the kinetic parameters by Lineweaver-Burk plots and the inhibition constants, a concentration of CO_2_ between 1.7 to 17 mM was used. At least six measurements of the original 5–10% reaction were used to assess the initial velocity for each inhibitor. The uncatalyzed rates were identically determined and detracted from the total observed rates. Stock inhibitor solutions (10–100 mM) were prepared in distilled-deionized water and dilutions up to 0.01 mM were done with the buffer test. Inhibitor and enzyme solutions were preincubated together for 15 min at room temperature prior to assay, in order to allow for the formation of the E-I complex or for the eventual active site mediated hydrolysis of the inhibitor. The inhibition constants were obtained by non-linear least-squares methods using PRISM 6 and the Cheng-Prusoff equation, as reported earlier^62–64^, and represent the mean from at least three different determinations. All CA isoforms were recombinant ones obtained in-house. All salts/small molecules were of the highest purity available, from Sigma-Aldrich (Milan, Italy).

## Results and discussion

The genome of diatoms encodes for a diversity of CA-classes, which are probably involved in the algal metabolism, pH regulation and balance of CO_2_/HCO_3_^–^ concentrations. In the marine diatom *Phaeodactylum tricornutum* five *α*-CAs have been identified, which are confined in the matrices of the four-layered plastid membranes^25^^,^^65^, together with two *β*-CAs (PtCA1 and PtCA2) located in the pyrenoid, two mitochondrial γ-CAs^25^, and one *θ*-CA in the lumen of the pyrenoid-penetrating thylakoid^25^. The genome of *Thalassiosira weissflogii* encodes for two CAs (TWCA1 and CDCA1) classified as the first members of the δ- and ζ-class, respectively^66^. Recently, as mentioned above, it has been demonstrated that the low-CO_2_-inducible protein (LCIP63) with the molecular weight of 63.0 kDa is a new class of CA (i.e., the ι-CA) and is present in the marine diatom *Thalassiosira pseudonana*. This CA showed peculiar characteristics: presence of an endoplasmic reticulum signal peptide (22 amino acid residues), and a chloroplast signal peptide (34 amino acid residues) at the N-terminal part. It is a multidomain protein with four repeated domains (multidomain protein), each of them homologous to the calcium/calmodulin dependent protein kinase II Association Domain. Furthermore, Mn(II) is reported to be the preferred catalytic ion cofactor^24^. LCIP63 homologues were found in other diatoms, algae, bacteria, and archaea^24^ and this prompted us to investigate the biochemical properties of the bacterial ι-CAs.

### Primary sequence and phylogenetic analysis

**Figure 1.**
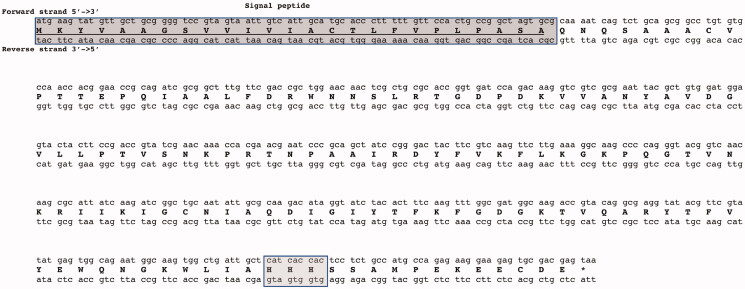
Graphical representation of the nucleotide sequence and translated amino acid sequence of BteCAι. The open reading frame (ORF) is indicated by bold capital letters, while the two nucleotide strands (5′ and 3′) by lower-case letters. The dark box (top of the figure) contains the amino acid residues identified as the protein signal peptide. The lightbox (bottom of the picture) contains the three histidines motifs, which is a unique characteristic of the ι-CA, and thus, not present in the other CA-classes reported up to now.

Using LCIP63 as query sequence and the similarity searching programme BLAST^67^, a ι-CA homologous to the LCIP63 was identified in the genome of the bacterium *Burkholderia territorii*. The bacterial ι-CA nucleotide sequence shows an open reading frame encoding for a polypeptide chain (BteCAι) of 162 amino acid residues (Figure 1), having a theoretical molecular mass of 17.8 kDa.

**Figure 2.**
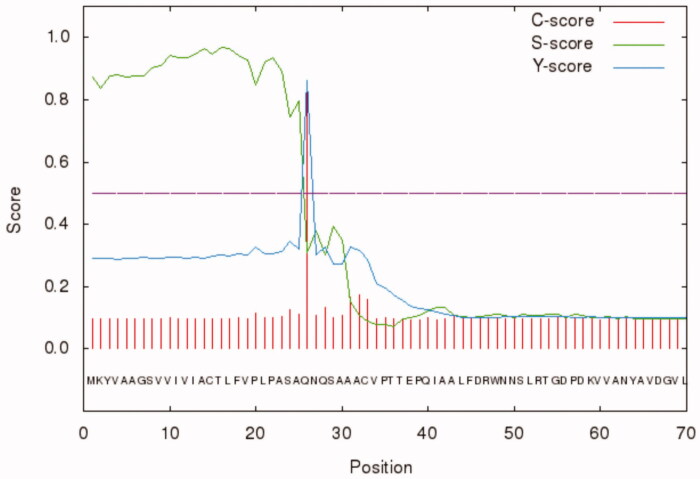
Graphical output generated by the software SignalP 4.1. The graph shows three different scores for the first 70 amino acid residues of BteCAι. Legend: X-axis, amino acid position; Y-axis, the following three scores: C-score (red line), raw cleavage site score; S-score (green line), signal peptide score; Y-score (blue line), combined cleavage site score.

Besides, BteCAι was investigated for the presence of the secretion signal peptide at the N-terminal using the bioinformatics tool “SignalP 4.1” (http://www.cbs.dtu.dk/services/SignalP/). As shown in Figure 2, the programme identified a putative signal peptide with a cleavage site between amino acid residues 25 and 26 (ASA-QN) of the BteCAι N-terminal amino acid sequence (Figure 2).

We should mention that a common feature of the bacterial *α*-CAs identified in the Gram-negative bacteria is the presence of a secretory signal peptide at the N-terminal of the amino acid sequence. The secretory signal peptide allows the periplasmic localisation of the *α*-CAs. In our investigation on the bacterial CAs, it has also been noted that *β*- or *γ*-CA primary structures belonging to some pathogenic Gram-negative bacteria are typified by a secretory signal peptide of 18 or more amino acid residues^7^^,^^8^. These findings coupled with the evidence that also the bacterial ι-CAs have a putative secretory signal peptide suggest that the *β*-, *γ*- and/or ι-CAs characterised by the presence of a signal peptide might have in the Gram-negative bacteria a periplasmic localisation supporting or complementing the same role of the *α-*CAs^7^^,^^8^.

**Figure 3.**
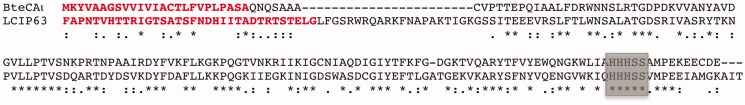
Alignment of BteCAι and LCIP63. The peptide signal (in red) are enclosed for both proteins. In the case of LCIP63, only the first domain of the four repeated domains was considered. The grey box represents the common consensus of the ι-CAs.

**Figure 4.**
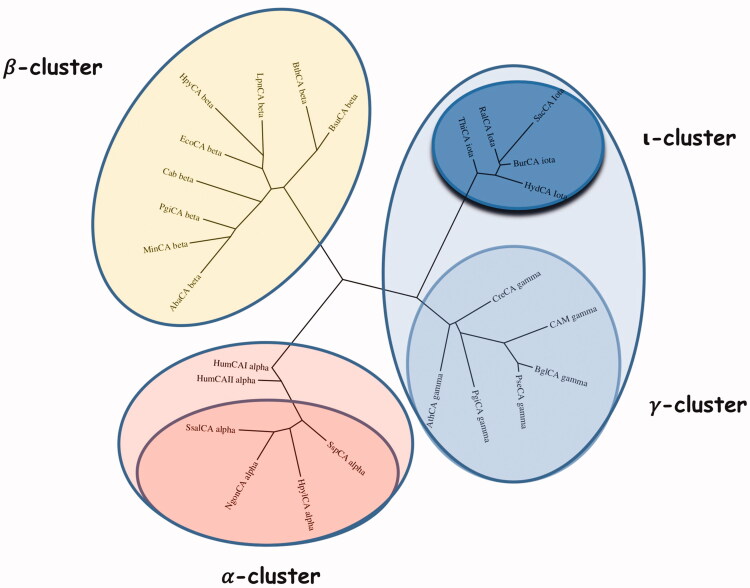
Phylogenetic analysis was carried out using the PhyML programme. The bootstrap consensus tree (100 replicates) was obtained using all four classes of CAs identified in the genome of different bacteria. The two human *α*-CA isoforms hCA I and II were included in the phylogenetic analysis, too. Legend: *α*-cluster (pink), *β*-cluster (yellow), *γ*-cluster (light blue), and ι-cluster (dark blue).

Figure 3 shows the alignment of the ι-CA amino acid sequences identified in *B. territorii* and *T. pseudonana*. BteCAι displayed a 35% identity with LCIP63 domain, and, as expected, showed the typical ι-CA consensus sequence “HHHSS” at the C-terminus of the polypeptide chain, which is a distinctive character of this new CA-class. Generally, the analysis of the sequence hallmarks is far from being exhaustive, as it does not take into account all the amino acid substitutions that differentiate this new class from the other bacterial CA classes. Hence, a phylogenetic tree has been constructed to better evidence the relationship of the bacterial ι-CA sequences with respect to the other bacterial CA-classes (*α*, *β*, and *γ*) (Figure 4). The two human isoforms hCA I and hCA II were added in this analysis, too. From the dendrogram shown in Figure 4, the bacterial ι-CAs clustered in a group closely associated with the bacterial *γ*-CAs. Although closer to the *γ*-CA, the ι-CA cluster appears well separated from them as well as distinct from all other CA-clusters. Thus, this finding reinforces the fact that these proteins were classified in a new CA class. We even speculate that the bacterial ι-CAs may be the result of a modification of an “ancestor gene” commune with that of the *γ*-CAs, which we demonstrated earlier to represent the ancestral class among all CA-classes^7^^,^^9^.

### Recombinant enzyme preparation and protonography

**Figure 5.**
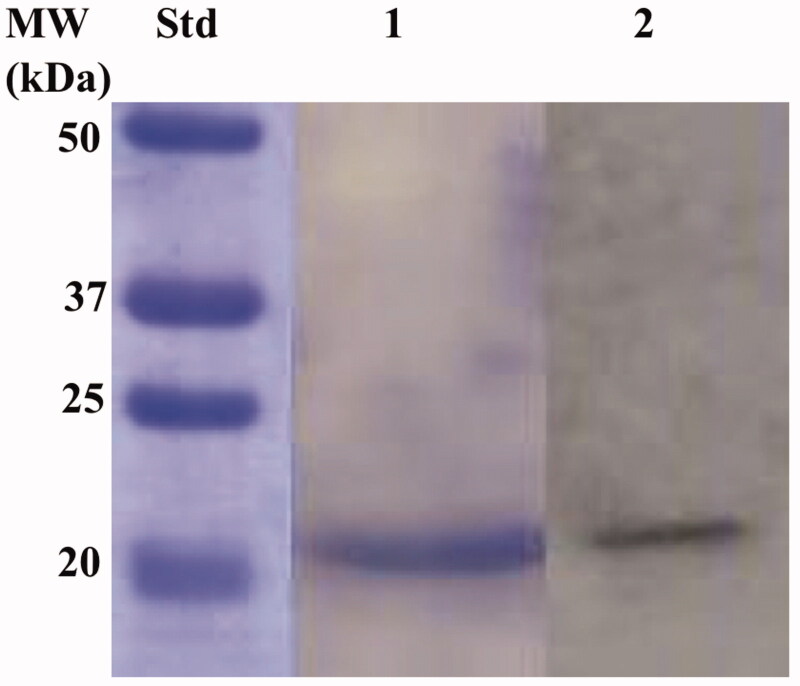
Combined lanes of SDS-Page and Western blot of BteCAι. The affinity purified recombinant BteCAι was subjected to SDS-PAGE (Lane 1) and then electro-blotted and incubated with the anti-HisTag (Lane 2, Panel A). Lane Std, molecular markers.

The recombinant BteCAι was produced as a fusion protein with a tail containing six histidines (His-Tag) at the N-terminal end, without its secretory signal peptide. The heterologous overexpression was induced by the addition of IPTG to the *E. coli* BL21 DE3 Codon plus cells. A supplement of 0.5 mM ZnCl_2_ was added to the host cells to allow the correct protein folding. Most of the ι-CA activity was recovered in the soluble bacterial cellular extract prepared by sonication and centrifugation. Using the affinity column (His-select HF Nickel affinity gel), the BteCAι fusion protein was purified to the homogeneity as a subunit with an apparent molecular weight of about 19,0 kDa as indicated by SDS-PAGE and Western Blot (Figure 5).

**Figure 6.**
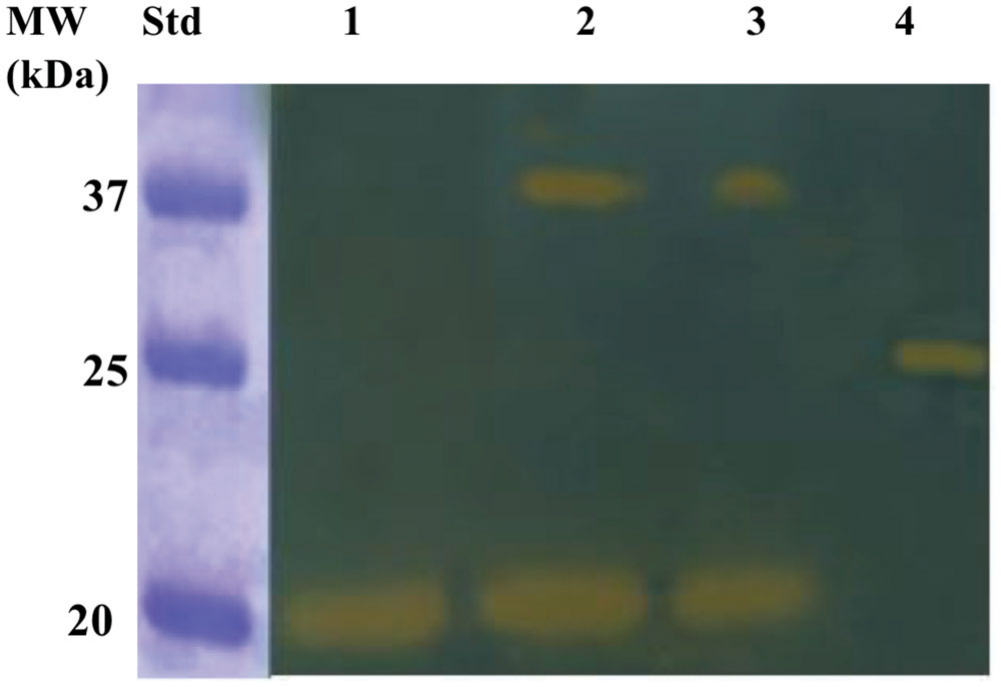
Developed protonogram showing the CO_2_ hydratase activity of BteCAι. The purified bacterial ι-CA was mixed with the Loading Solution Buffer (LSB) containing SDS at different concentrations (1.0, 0.5 and 0.1%) and loaded on the gel at 10 µg/well. The yellow bands correspond to the enzyme activity responsible for the drop of pH from 8.2 to the transition point of the dye in the control buffer. Legend: Lane 1, BteCAι with 1% SDS (protein in a monomeric state, MW: 19.0 kDa); Lane Std, Molecular markers. Lane 2 and 3 purified BteCAι mixed with 0.5 and 0.1% SDS, respectively (monomer and dimer); Lane 4, commercial bovine CA, used as positive controls.

The purified BteCAι was investigated for its catalytic activity on the polyacrylamide gel through the use of a technique developed in our laboratories, the so-called protonography^56^. The method allows the monitoring of the pH variation in the polyacrylamide gel due to the CA-catalysed conversion of CO_2_ to bicarbonate and protons. Besides, to investigate the oligomeric protein state, aliquots of the purified BteCAι were mixed with a loading solution buffer (LSB) containing different concentrations of SDS, ranging from 1.0 to 0.1%. The resulting protonogram shown in Figure 6 evidenced the yellow bands due to the production of ions (H^+^) during the CO_2_ hydration reaction. The protonogram shows that the ι-CA is present only in the monomeric state when BteCAι was treated with LSB containing 1% of SDS. When the concentration of SDS in the LSB is less than 1% (0.5 or 0.1%), it was possible to see the two oligomeric states of the enzyme with an apparent molecular weight of 19.0 and 40 kDa, which correspond to the monomer and dimer form, respectively (Figure 6). Of course, with the aid of protonography is not possible to establish if the ι-CA monomer can perform the hydration reaction since the protonography technique requires the SDS removal from the gel.^56^ This procedure potentially led to the rearrangement of ι-CA monomers in the polyacrylamide gel, reconstituting the dimeric form of the enzyme. Thus, if the monomer is inactive, we always will see the presence of a yellow band due to the reconstituted dimer at the position of the ι-CA monomers. Thus, the activity of the ι-CA dimer can be explain by the two following conditions: (1) the ι-CA dimer is subject to a structural arrangement of the ι-CA monomers similar to that observed in the *β*- and γ-CAs, whose monomers assemble into dimer (*β*-CA) or trimer (γ-CA) to produce the catalytic active form of the enzyme^29^^,^^68^; (2) the ι-CA monomer is active as reported for the bacterial *α*-CAs, which crystallise as a dimer^33^.

### Enzyme activity and kinetic parameters

The CO_2_ hydratase activity and the kinetic constants of the purified and soluble BteCAι were determined using the stopped-flow technique. The enzyme had a high catalytic activity for the physiological reaction of CO_2_ hydration to bicarbonate and protons, with a k_cat_ 3.0 × 10^5 ^s^−1^ and a catalytic efficiency (k_cat_/K_M_) of 3.9 × 10^7^ M^−1^ s^−1^, and was also sensitive to inhibition by the sulphonamide acetazolamide (K_I_ = 64.9 nM). The BteCAι kinetic parameters are shown in Table 1 and compared with those of the *α*-CAs from the *Homo sapiens* (isoforms hCAI and hCAII) as well as with a representative belonging to the bacterial *α*, *β*, and *γ*-class.

**Table 1.** BteCAι kinetic parameters compared with those calculated for the two human isoforms hCA I and II (*α*-class), and the *α*-, *β*-, *γ*- and ι-CAs from different bacterial species (all of them with Zn(II) at the active site).

Organisms	Acronym	Class	K_cat_ (s^–1^)	k_cat_/K_M_ (M^–1^·s^–1^)
*Homo sapiens*	hCA I^a^	*α*	2.0 × 10^5^	5.0 × 10^7^
	hCA II^a^	*α*	1.4 × 10^6^	1.5 × 10^8^
*Vibrio cholerae*	VchCA^b^	*α*	8.23 × 10^5^	7.0 × 10^7^
*Burkholderia pseudomallei*	Bps *β*CA^c^	*β*	1.6 × 10^5^	3.4 × 10^7^
*Burkholderia pseudomallei*	Bps*γ*CA^d^	*γ*	5.3 × 10^5^	2.5 × 10^7^
*Burkholderia territorii*	BteCAι^e^	ι	3.0 × 10^5^	9.7 × 10^7^

The CO_2_ hydration reaction was followed at 25 °C, in 20 mM Tris buffer and 20 mM NaClO4, pH 8.3. ^a^From ref. 3; ^b^From ref. 28; ^c,d^From ref. 10; ^e^This work.

BteCAι k_cat_ value is similar to that obtained for the other bacterial CAs, as well as for the hCA I. These results are of extreme importance in the field of the inhibition of the bacterial CAs, which are crucial molecules for supporting the microbial functions involving dissolved inorganic carbon. Thus, among the new antibacterial should also be considered those new inhibitors able to inhibit the activity of the bacterial i-CA for contrasting the bacterial growth and their virulence.

### Catalytic ion cofactor

**Figure 7.**
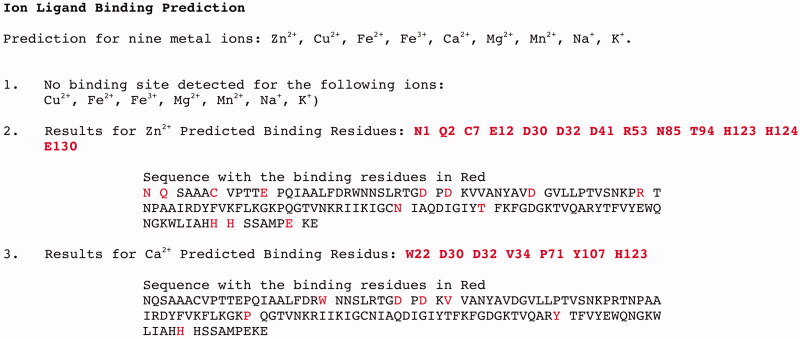
Output generated by the bioinformatics programme IonCom using as query sequence the BteCAι amino acid polypeptide chain. The output evidenced the ions not detected for the binding (point 1); and the possible ions, such as Zn^2+^ and Ca^2+^ (points 2 and 3). Besides, it reports the BteCAι amino acid sequence with the binding residues indicated in red.

As described above, the recombinant BteCAι was overexpressed to supplement the bacterial culture medium with 0,5 mM of ZnCl_2_. Following the strategy proposed by Jensen et al.^24^, the amino acid sequence of BteCAι was submitted to a bioinformatics programme able to predict the binding of nine metal ions (Zn^2+^, Cu^2+^, Fe^2+^, Fe^3+^, Ca^2+^, Mg^2+^, Mn^2+^, Na^+^, K^+^) with the query sequence protein (BteCAι) (https://zhanglab.ccmb.med.umich.edu/IonCom/)^69^. The output generated by the programme is shown in Figure 7.

The programme predicted that the recombinant BteCAι could bind only Zn^2+^ and Ca^2+^. To verify this, an aliquot of BteCAι was treated with EDTA overnight. The treatment with EDTA inactivated the enzyme as reported for the other CAs having Zn^2+^ as a catalytic cofactor. Intriguing, the addition of Zn^2+^ or Ca^2+^ fully restored the enzyme activity (Table 2). We also try to restore the enzyme activity using a metal, which was not predicted by the programme IonCom, such as Mn^2+^. From Table 2, it is readily apparent that this ion did not restore the enzyme activity, diversely as described for the ι-CA identified in the genome of the marine diatom *T. pseudonana*^24^.

**Table 2.** Effect of the addition of the Zn^2+^, Ca^2+^, and Mg^2+^ on the activity of the EDTA-treated BteCAι.

BteCAι + EDTA (WAU/mg)	Addition of the ion cofactor (WAU/mg)		
	Zn^2+^	Ca^2+^	Mg^2+^
0	343 ± 18	328 ± 21	0

### Homology modelling

**Figure 8.**
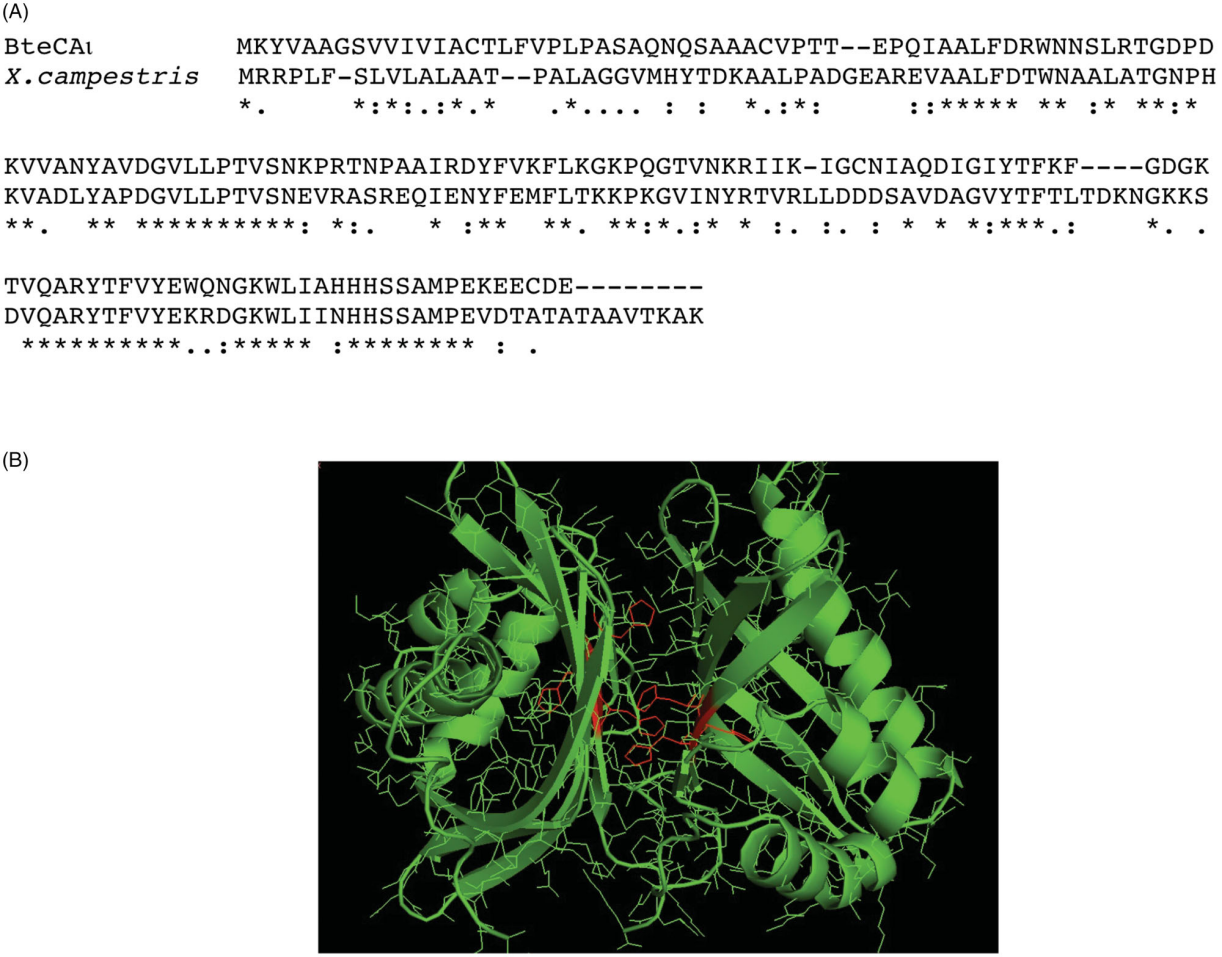
Panel A: Alignment of the amino acid sequences from B. territory and X. campestris. Panel B: Model of the bacterial BteCAι generated using the automated procedure on the server SWISS-MODEL (https://swissmodel.expasy.org).

To date, no X-ray crystallographic or NMR structures are available for ι-CA and, as obtaining them is an intricate and time consuming process. However, the computational approach to predict protein three-dimensional structures starting from the amino acid sequence is an appealing alternative. Thus, using a fully automated protein homology modelling server SWISS-MODEL (https://swissmodel.expasy.org), we generated a raw first model of the bacterial BteCAι. The automated mode selects the structural templates that maximise the expected quality of the model. Two main templates were identified with a 50% identity (the other templates showed an identity ranging from 16 to 27%). Surprisingly, all the templates were identified as putative calcium/calmodulin-dependent protein kinase II Association Domain. We focalised our attention on the first template homologous to BteCAι and coming from *Xanthomonas campestris*, a bacterial species that causes a variety of plant diseases^70^. Its PDB code is 3h51, while NP_636218.1 is the protein accession number. Astonishingly, the code NP_6362218.1 (now WP_011036063.1 in the Pubmed (https://www.ncbi.nlm.nih.gov/protein/WP_011036063.1)) corresponded to a sequence classified as SgcJ/EcaC family oxidoreductase, which is the annotation of most of the sequences found during the search of the bacterial ι-CAs in the data bank. This prompt us to align the amino acid sequence of BteCAι with that identified in *X. campestris* (Figure 8, Panel A). The ι-CA amino acid sequences identified in *B. territorii* displays a 42% identity with the *X. campestris* amino acid sequence (38%identity with LCIP63 domain), and, as expected, the typical ι-CA consensus “HHHSS” at the C-terminus of the polypeptide chain is present (Figure 8, Panel A). In Figure 8, Panel B, we report the generated model of BteCAι with the consensus histidines of each monomer highlighted in red. We want stress that the resulting model predicted a dimeric organisation of the protein. This confirms the results obtained from the protonography performed at different concentration of SDS, which evidenced the presence of an hydratase activity at a molecular weight of 40.0 kDa (enzyme dimeric state). Of course, work is in progress for obtaining the crystal structure of BteCAι especially for understanding the coordination of the catalytic ion cofactor, if Zn^2+^, Mn^2+^ or another metal ion, as well as the metal coordination pattern.

## Conclusions

The analysis of BteCAι primary structure evidenced a secretory signal peptide consisting of 25 amino acid residues at the N-terminal part of the protein. The phylogenetic analysis carried out using bacterial *α*-, *β*-, γ-, and ι-CAs evidenced that the new ι-CAs were closer to the γ-classes than the other bacterial CAs (*α* and *β*). The determination of the kinetic constants showed that the bacterial ι-CA is a good catalyst for the hydration of CO_2_ to bicarbonate and protons, with a k_cat_ of 3.0 × 10^5^ s^−1^ and k_cat_/K_M_ of 3.9 × 10^7^ M^−1^ s^−1^, and is also sensitive to inhibition by the sulphonamide acetazolamide (K_I_= 64.9 nM). With the aid of the protonography, a technique developed by our groups, which allows the detection of the hydratase activity onto a polyacrylamide gel, it has been demonstrated that BteCAι can be present as a dimer. The structure prediction model showed that BteCAι is formed by two equivalent monomers, which appears as a “butterfly” structure where the Zn^2+^ ion cofactor of the active site might be coordinated by two histidines of a monomer and one histidine of the other monomer. Of course, the metal coordination will be thoroughly investigated with the resolution of the three-dimensional structure of this new class of CA, which is in progress in our laboratories.
